# High prevalence and diversity of *Toxoplasma gondii* DNA in feral cat feces from coastal California

**DOI:** 10.1371/journal.pntd.0011829

**Published:** 2023-12-15

**Authors:** Sophie Zhu, Lauren Camp, Anika Patel, Elizabeth VanWormer, Karen Shapiro

**Affiliations:** 1 Department of Pathology, Microbiology, and Immunology, School of Veterinary Medicine, University of California, Davis, Davis, California, United States of America; 2 Veterinary Medical Teaching Hospital, School of Veterinary Medicine, University of California, Davis, Davis, California, United States of America; 3 School of Veterinary Medicine and Biomedical Sciences, University of Nebraska, Lincoln, Nebraska, United States of America; 4 School of Natural Resources, University of Nebraska, Lincoln, Nebraska, United States of America; Universidade Federal de Uberlandia, BRAZIL

## Abstract

*Toxoplasma gondii* is a zoonotic parasite that can cause severe morbidity and mortality in warm-blooded animals, including marine mammals such as sea otters. Free-ranging cats can shed environmentally resistant *T*. *gondii* oocysts in their feces, which are transported through rain-driven runoff from land to sea. Despite their large population sizes and ability to contribute to environmental oocyst contamination, there are limited studies on *T*. *gondii* oocyst shedding by free-ranging cats. We aimed to determine the frequency and genotypes of *T*. *gondii* oocysts shed by free-ranging domestic cats in central coastal California and evaluate whether genotypes present in feces are similar to those identified in sea otters that died from fatal toxoplasmosis. We utilized a longitudinal field study of four free-ranging cat colonies to assess oocyst shedding prevalence using microscopy and molecular testing with polymerase chain reaction (PCR). *T*. *gondii* DNA was confirmed with primers targeting the ITS1 locus and positive samples were genotyped at the B1 locus. While oocysts were not visualized using microscopy (0/404), we detected *T*. *gondii* DNA in 25.9% (94/362) of fecal samples. We genotyped 27 samples at the B1 locus and characterized 13 of these samples at one to three additional loci using multi locus sequence typing (MLST). Parasite DNA detection was significantly higher during the wet season (16.3%, 59/362) compared to the dry season (9.7%; 35/362), suggesting seasonal variation in *T*. *gondii* DNA presence in feces. High diversity of *T*. *gondii* strains was characterized at the B1 locus, including non-archetypal strains previously associated with sea otter mortalities. Free-ranging cats may thus play an important role in the transmission of virulent *T*. *gondii* genotypes that cause morbidity and mortality in marine wildlife. Management of free-ranging cat colonies could reduce environmental contamination with oocysts and subsequent *T*. *gondii* infection in endangered marine mammals and people.

## Author summary

*Toxoplasma gondii* is a parasite that can cause severe disease and death in warm-blooded animals such as humans and sea otters. Feral cats can release *T*. *gondii* oocysts, or environmentally resistant ‘eggs’, in their feces. These oocysts can be transported by surface runoff after rainfall and go on to contaminate bodies of water. Oocyst shedding from feral cats is not well understood, and whether they shed specific types of *T*. *gondii* that have been found in sea otters that died from toxoplasmosis is not known. We aimed to determine how often feral cats in central coastal California shed *T*. *gondii* oocysts and identify types of the parasite in feces. Although we did not see oocysts, 25.9% of samples tested positive for *T*. *gondii* DNA. Parasite DNA was more common during the wet vs. dry season. There were diverse *T*. *gondii* types found in feral cat feces, including types that were previously found in sea otters that died from toxoplasmosis. Future management of feral cats could reduce environmental contamination and in turn, *T*. *gondii* infections in sea otters and humans.

## Introduction

*Toxoplasma gondii* is a zoonotic apicomplexan parasite that infects warm-blooded vertebrates and causes morbidity and mortality in many species, including people and marine mammals. *T*. *gondii* has three primary life stages: tachyzoites, which propagate infection within the host; bradyzoites, which reside in tissue cysts during chronic infections; and sporozoites contained within oocysts, the environmental stage of the parasite [1]. *T*. *gondii* can only sexually reproduce in the intestine of its definitive hosts, domestic and wild felids, which are the only animals that shed oocysts in their feces. Oocysts are environmentally resistant and, when sporulated, can withstand extended exposure to high and low temperatures and UV from sunlight [2,3]. Surface runoff can mobilize *T*. *gondii* oocysts from soil into oceans or other bodies of water [4]. Waterborne oocysts can remain in the water column or may bioaccumulate in invertebrates such as mussels, snails, and oysters [5–7]. Marine mammals can directly or indirectly ingest oocysts from one of these pathways and become infected with *T*. *gondii*, which may lead to severe disease or even death [8].

In the 1990s, researchers discovered high numbers of fatal toxoplasmosis cases in the endangered southern sea otter (*Enhydra lutris nereis*) in California [8], and subsequent studies found that diet choice [9] and living near freshwater runoff [4,10] are risk factors for *T*. *gondii* infection. More recently, studies identified specific strains associated with severe disease and mortality in sea otters [11]. Despite this rich body of research on *T*. *gondii* infection in otters, critical information about the terrestrial origins of virulent *T*. *gondii* genotypes is lacking. Even though both domestic and wild felids can carry and shed *T*. *gondii*, domestic cats may contribute more to environmental oocyst load due to their large population sizes [12]. However, there are limited studies of oocyst shedding in free-ranging cats, none over a long period, and few that report the genotypes of *T*. *gondii* detected in feces. Since oocysts drive *T*. *gondii* infections [13], documenting shedding patterns in a natural setting is crucial to understanding oocyst exposure risk over time.

Genotype is an important factor that may be associated with the outcome of infection in sea otters [14]. Non-archetypal genotypes, namely Type X (ToxoDB #5) or X variants, were previously detected in otters that died from toxoplasmosis as a primary cause of death and from tissues of terrestrial domestic and wild felids [14]. Though the land-to-sea transmission of *T*. *gondii* is established, the degree to which feral outdoor cats shed this genotype is unknown. While non-archetypal *T*. *gondii* genotypes are found in domestic cats and wild felids [15–17], most molecular studies have focused on characterizing parasite DNA from the tissues of felids (bradyzoite cysts)–rather than oocysts from feces. Mixed *T*. *gondii* infections in cats (infection with more than one *T*. *gondii* strain) could result in different genotypes detected in tissue cysts as compared with genotypes of oocysts shed in feces. Mixed infections with different genotypes have been identified in wild and domestic felids [18,19], and co-infection may be a common occurrence if these hosts encounter multiple *T*. *gondii* genotypes from the environment and infected prey throughout their lifetime. Detection of non-archetypal genotypes of *T*. *gondii* from free-ranging domestic cat feces would further enhance understanding of the role these hosts play in land-to-sea transmission of virulent strains of *T*. *gondii* to marine environments.

Our initial objectives were to 1) determine the presence and prevalence of *T*. *gondii* oocyst shedding in free-ranging cat feces over time; and 2) characterize genotypes of *T*. *gondii* in free-ranging cat feces. We hypothesized that free-ranging cats shed *T*. *gondii* oocysts and that shedding prevalence would be higher in the wet versus dry season. We also hypothesized that non-archetypal genotypes of *T*. *gondii* previously isolated from dead sea otters would be detected in free-ranging cat feces. Identifying the most relevant populations of cats that contribute virulent genotypes and when these oocysts enter the environment will allow for targeted management efforts to protect susceptible wildlife and human health. As we discuss below, not all objectives were fulfilled due to challenges with visualization of oocysts using microscopy.

## Methods

### Site identification and fecal collection

We identified four free-ranging cat colonies within watersheds bordering Monterey Bay coastal waters based on data from a previous study [20] and personal correspondence with animal control personnel from organizations near Santa Cruz and Monterey, CA. The four colonies were in Gilroy (Santa Clara County), Moss Landing (Monterey County), Aptos (Santa Cruz County), and Santa Cruz (Santa Cruz County). Three sites were located directly on the coastline, and one (Gilroy) was located inland (25.4 km from coast) in a watershed which drains directly into Monterey Bay. The Monterey Bay area was selected as a relevant study region because it provides critical habitat to threatened southern sea otters where infection with *T*. *gondii* is common [11]. Monthly sampling at cat colony latrines was conducted every four to five weeks between July 2020 and August 2022 (24 months). Sampling did not occur in February or March 2021 due to COVID-19 restrictions, and sampling occurred twice in April 2022 due to the low sample size (n = 5) during the first collection trip earlier that month. Latrines/defecation sites were identified from extensive visual screening near the colony, starting with areas near feral cats (if present), close to feeding sites (if present), and in areas with sandy sediment that are likely to be favored by cats as defecation sites (if present). Sampling and observation were always conducted between 6 and 10AM PST for approximately 45 minutes to one hour per site to increase likelihood of spotting cats before parks became busier with human activity. We collected samples identified as domestic cat feces within approximately a 500-meter radius of known cat sightings or feeding stations in fecal collection cups, transported them in a cooler with cold packs to UC Davis, and refrigerated them until processing the following day. Feces was identified as being from domestic cats based on visual presence of cats in the area, proximity to any feeding sites, and size and shape of the fecal matter [21]. In addition to sample collection, we recorded the date of collection, sampling site, the number of adult cats seen (if present), the number of kittens seen (if present), and notes on location conditions (i.e., flooding, overgrown brush, etc.) during each monthly sampling trip.

### Sample size calculation

We determined the sample size for detection of disease (*T*. *gondii* oocyst shedding) in a large population, assuming test sensitivity of 90% and test specificity of 100%. The ability for *T*. *gondii*-like oocysts to be confirmed as *T*. *gondii* using PCR-based analysis and sequencing supports the assumed test specificity of 100%. Although the test sensitivity for detecting *T*. *gondii*-like oocysts using flotation and microscopy has not been reported, we detected oocyst shedding in experimentally and naturally infected domestic cats in previous studies with this approach [12,22]. In previous cross-sectional studies, ~2% of free-ranging domestic cats were shedding *T*. *gondii*-like oocysts at a given time [12]. Only half of those oocysts were confirmed as *T*. *gondii* using PCR, giving a conservative estimated shedding prevalence of 1%. Using power size calculations, a minimum of 332 fecal samples would be necessary to detect a shedding prevalence of 1% with the desired probability of 0.95 [23].

### Fecal flotation

We used double centrifugation flotation to process fecal samples and examine specimens for *T*. *gondii* oocysts using both brightfield and UV epifluorescence at 100X magnification. Oocysts that were morphologically similar to those of *T*. *gondii* oocysts were examined and measured at 200X, however the unsporulated and sporulated coccidian oocysts observed were determined to be within the size range reported for *Cystoisospora* spp. (larger than *T*. *gondii* which should measure 10X12 μm). Two individuals screened each slide to improve the sensitivity of our microscopic examination, including diagnostic parasitologists with extensive experience identifying parasite ova and oocysts (L. Camp or K. Shapiro). Flotations were performed with zinc sulfate solution (specific gravity (SG) 1.18) for the first six months of sampling (August 2020-January 2021) and subsequently (February 2021-August 2022) using a sucrose solution (SG 1.18) because crystal formation of zinc sulfate hindered screening when multiple personnel examined slides over time. All parasite ova were recorded when seen and categorized according to size and morphology (i.e., *T*. *gondii*-like, *Cystoisospora* spp.-like, *Toxocara* spp.-like, *Sarcocystis* spp.-like). After microscopic examination, the cover slip was removed and rinsed with 20–25 mL of DI water into a clean 50 mL falcon tube and centrifuged at 1500 X g to obtain a 100–500 *μ*L pellet for nucleic acid extraction and PCR. Testing flotation material for *T*. *gondii* DNA enabled us to target a matrix with higher likelihood of parasite detection and reduced interference from fecal debris [24]. The detection limit for *T*. *gondii* DNA detection using a similar approach and extraction kit followed by nested PCR has been reported to be 5 oocysts/250 mg spiked feces [25]. Cover slips were not retained for all samples during the first three months of sampling (July—September 2020) due to lack of *T*. *gondii* oocyst visualization, however we began screening all samples with PCR from October 2020 onward as an amendment to our initial methodology.

### DNA extraction, nested conventional PCR, and RT-qPCR

We centrifuged pellets obtained from washing fecal flotation coverslips at 2000 x g for 10 minutes and processed 100 *μ*l for nucleic acid extraction according to the manufacturer’s instructions for the DNeasy Blood and Tissue Kit (QIAGEN). Additionally, we performed a 4-minute freeze (liquid nitrogen -196°C), and a 4-minute thaw (boiling water, 100°C) cycle to rupture oocyst walls before adding proteinase K [26]. A negative extraction control was included in each batch to monitor for potential contamination. For initial screening of samples for apicomplexan parasite DNA, we used a conventional nested PCR targeting the first internal transcribed spacer 1 (ITS-1), located between 18S and 5.8S rRNA genes of the small subunit ribosomal RNA (SSU rRNA) [27]. The PCR reaction consisted of 18.6 *μ*l (internal) or 21.6 *μ*l (external) of DNase- and RNase-free distilled water, 0.5 *μ*l each of 50 *μ*M forward and reverse primers, 0.8 *μ*l of 10% Bovine Serum Albumin, 25 *μ*l of 2X Amplitaq Gold 360MM, and 5 *μ*l (external) or 2 *μ*l (internal) of DNA template. Each PCR run contained one positive control (DNA derived from *Sarcocystis neurona* merozoites) and three negative controls (extraction reagents with distilled water, PCR reagent control, and another PCR reagent control with distilled water added in the nested reaction step). PCR amplification products were separated and visualized with gel electrophoresis on a 2% agarose gel stained with Red Safe and viewed with a UV transilluminator. Samples that yielded no DNA amplification were run twice more for a total of triplicate attempts to increase the sensitivity of *T*. *gondii* DNA detection. Samples with amplified DNA consistent with positive control bands were cut from the gel, purified using the Qiagen Qiaquick Gel Extraction kit, and submitted for Sanger sequencing at the UCDNA Sequencing Facility to confirm molecular identity.

Since detecting *T*. *gondii* DNA is not indicative of parasite stage, and because previous studies have shown that bradyzoite DNA from infected prey can be detected from cat feces [28], we attempted to differentiate oocyst messenger RNA (mRNA) vs bradyzoite DNA using RT-qPCR targeting SporoSAG mRNA (SporoSAG is a surface antigen glycoprotein found on the surface of sporozoites, the parasite stage contained within *T*. *gondii* oocysts) [29]. Starting in March 2022, we tested fecal samples that were confirmed to have *T*. *gondii* DNA (based on conventional PCR and sequencing at the ITS-1 locus) by extracting mRNA and performing Reverse-transcription qPCR (RT-qPCR) following previously described and validated methods for detection of SporoSAG mRNA in oocysts [29]. mRNA was extracted using the Dynabeads mRNA DIRECT Kit (Invitrogen, CA, USA) according to the manufacturer’s instructions with slight modifications [29,30]. Samples were heated at 45°C for 20 minutes to induce mRNA expression, and then 200 *μ*L of Lysis-Binding buffer was added followed by six freeze/thaw cycles using liquid nitrogen (1 min.) and a 65°C water bath (1 min.). Oligo (dT)_25_ magnetic beads (40 *μ*L) from the Dynabeads mRNA DIRECT Kit were added to each sample and left to incubate at 15 rpm on a rotator at room temperature for 20 minutes to allow for mRNA-bead attachment. The magnetic beads were separated from the supernatant using a magnet and then washed with 500 *μ*L of buffers A and B sequentially. Fifty *μ*L of cold elution buffer (10 mM Tris-HCL pH 7.5) were added and left to incubate at 80°C for 2 minutes [29]. After incubation, the tubes were immediately placed on the magnet, and supernatant containing mRNA was recovered. RT-qPCR was then performed using the QuantStudio 3 real-time PCR Instrument (Applied Biosystems, CA, USA). Each reaction mixture contained 5 *μ*l of TaqMan Fast Virus 1-step Master Mix, 2 *μ*L of a primer and probe mix (20X), 5 *μ*L of sample, and 8 *μ*L of water for a total volume of 20 *μ*L. Cycling conditions were set at 50°C for 10 min, 95°C for 20 s, followed by 40 cycles of 95°C for 3 s and 60°C for 30 s. A *T*. *gondii* oocyst mRNA control, negative extraction control, and non-template control (nuclease-free water) were included for quality control.

### Sequence analysis and *T*. *gondii* genotyping

Samples that were confirmed to contain *T*. *gondii* DNA by conventional PCR at the ITS-1 locus were further processed for *T*. *gondii* genotyping at the polymorphic B1 locus. The targeted multi-copy gene for genotyping (B1) was selected due to its sensitivity, specificity (no cross amplification with closely related apicomplexan parasites such as *Neospora* or *Sarcocystis*), and ability to discriminate among *T*. *gondii* genotypes [31, 32]. Nested PCR was performed using the same conventional PCR conditions except for including nested primers targeting the B1 locus, as previously described [7]. We attempted to amplify DNA in triplicate to maximize likelihood of DNA characterization at this locus. We purified PCR products from amplified samples using the QIAquick Gel Extraction Kit (QIAGEN) and submitted them for sequencing at the UC Davis ^UC^DNA Sequencing Facility following previously published protocols [7, 19]. Using Geneious software (R11 Biomatters Ltd., Auckland, New Zealand), we aligned the forward and reverse sequences and compared the consensus sequence with the GenBank database using the Basic Local Alignment Search Tool, BLAST (http://blast.ncbi.nlm.nih.gov/Blast.cgi) and the following reference strains: Type I (ATCC RH strain), II (ATCC ME49 strain), III (ATCC CTG strain), X (KM243033 Bobcat strain), X variant (sea otter MK988572 strain), and CR34 (mussel KM243033 strain). We further performed multi-locus sequence typing (MLST) at 12 polymorphic loci (SAG1, 5’-SAG2, 3’SAG2, alt. SAG2, SAG3, BTUB, GRA6, C22-8, C29-2, L358, PK1, Apico) on samples that amplified at the B1 locus using previously published protocols and re-ran samples that did not amplify in duplicate for a total of triplicate attempts for each sample [33]. Samples that exhibited novel single nucleotide polymorphisms (snps) were re-amplified if sufficient DNA remained and re-sequenced to confirm polymorphic nucleotides. For quality control, PCR reagent controls with and without added PCR-grade water were used with each run, as well as tachyzoite-derived *T*. *gondii* (RH strain Type I) as a positive control.

Parasite genotype was first determined via virtual restriction fragment length polymorphism (RFLP) at the B1 locus using Geneious software. To identify a specific SNP location in sequences that did not align at 100% identity with previously reported sequences, we then performed a local alignment in Geneious using common reference Types as described above and their associated GenBank accession numbers. After comparing with reference strains, an RFLP genotype and variant specific B1 sequence/strain type were assigned [31–33].

### Statistical analysis

Association between presence of any parasite ova during microscopy and *T*. *gondii* DNA detection in feces was evaluated using the Chi-square test, and 95% confidence intervals for proportions were calculated using the Wilson score interval. We performed statistical analysis in R version 3.6.1 [34] and carried out spatial visualization using the *ggplot2*, *rnaturalearth*, and *ggspatial* packages [35–37]. Due to the small number of spatial clusters (n = 4), we analyzed our data with a Bayesian approach to reduce bias in estimating parameter values using the package *brms* [38]. Season of collection (wet/dry), sampling site, kitten presence at sampling site, number of kittens, number of adult cats at specific sampling sites, and year were considered as explanatory variables in our models. A value of p<0.15 in univariable Chi-square or ANOVA tests was used as a threshold for variable inclusion into multivariable Bayesian models. Because the number of kittens was not significant using the aforementioned criteria, it was not considered in further model building.

We fit models for *T*. *gondii* DNA detection in feces with the season (wet/dry) of collection, kitten presence at the sampling site, and the number of adult cats at specific sampling sites as fixed effects, and year and site as random effects using a Bayesian mixed-effects generalized linear regression model with a binomial distribution. A null model with only random effects (year and site) was compared to fuller models with one or more additional variables. The final model was selected based on the leave-one-out cross-validation (LOO) information criterion and included all variables. Effective sample sizes from the posterior distribution ranged from 215 to 1474, and all predictors had R-hat values <1.01, indicating model convergence [38]. We used minimally informative priors for fixed effects (season, kitten presence, number of adults) to avoid overfitting; a normal distribution centered at 0 and a standard deviation of 10. A minimally informative prior was chosen over a non-informative prior to obtain more conservative estimates with consideration of the sampling scheme of our study [39]. Total monthly rainfall was used to characterize wet and dry seasons based on inches of precipitation at each sampling site measured by the University of California Santa Cruz Cooperative Extension between 2020 and 2022. The wet season began in any fall month (October-December) with more than 0.10 inches of rain and extended until the first month when spring rainfall fell below 0.10 inches. The dry season included all months outside of these criteria.

## Results

Over 24 months, we collected 404 fecal samples across four sites; 126 from Gilroy, 152 from Moss Landing, 68 from Aptos, and 58 from Santa Cruz (Table 1 and S1 Fig) [40]. No *T*. *gondii*-like oocysts were seen using microscopy, while ova of helminths such as *Toxocara cati*, and oocysts of other apicomplexan parasites (*Cystoisospora* spp., and *Sarcocystis* spp.) were visualized (S2 Table). *T*. *gondii* DNA was detected in 94 fecal samples and confirmed at the ITS-1 locus with 100% identity when compared with *T*. *gondii* reference strains. There was no association between visualizing *Cystoisospora* spp. (p = 1), *Sarcocystis* spp. (p = 1), or *Toxocara cati* (p = 0.91) during microscopic examination and *T*. *gondii* DNA detection.

**Table 1:** Microscopy and DNA detection prevalence of *T*. *gondii* in feces from free-ranging feral domestic cats in central coastal California.

Site	*T*. *gondii-*like oocyst prevalence [95% CI]	Fecal DNA prevalence [95% CI]	Dry season DNA prevalence [95% CI]	Wet season DNA prevalence [95% CI]
Gilroy	0/126 (0% [0–2.9])	36/111(32.4%[24.4‐41.6])	15/66 (22.7% [14.3–34.2])	21/45 (46.7% [32.9–61])
Moss Landing	0/152 (0% [0–2.5])	27/139 (19.4% [13.7–26.8])	11/66 (16.7% [9.6–27.4])	16/73 (21.9% [14–32.7])
Aptos	0/68 (0% [0–5.3])	17/61 (27.9% [18.2–40.2])	6/34 (17.6% [8.3–33.5])	11/27 (40.7% [24.5–59.3])
Santa Cruz	0/58 (0% [0–6.2])	14/51 (27.5% [17.1–41])	3/27 (11.1% [3.9–28.1])	11/24 (45.8% [27.9–64.9])
Total	0/404 (0% [0–0.9])	94/362 (25.9% [21.7–30.7])	35/193 (18.1% [13.3–24.2])	59/169 (34.9% [28.1–42.4])

**Fig 1:**
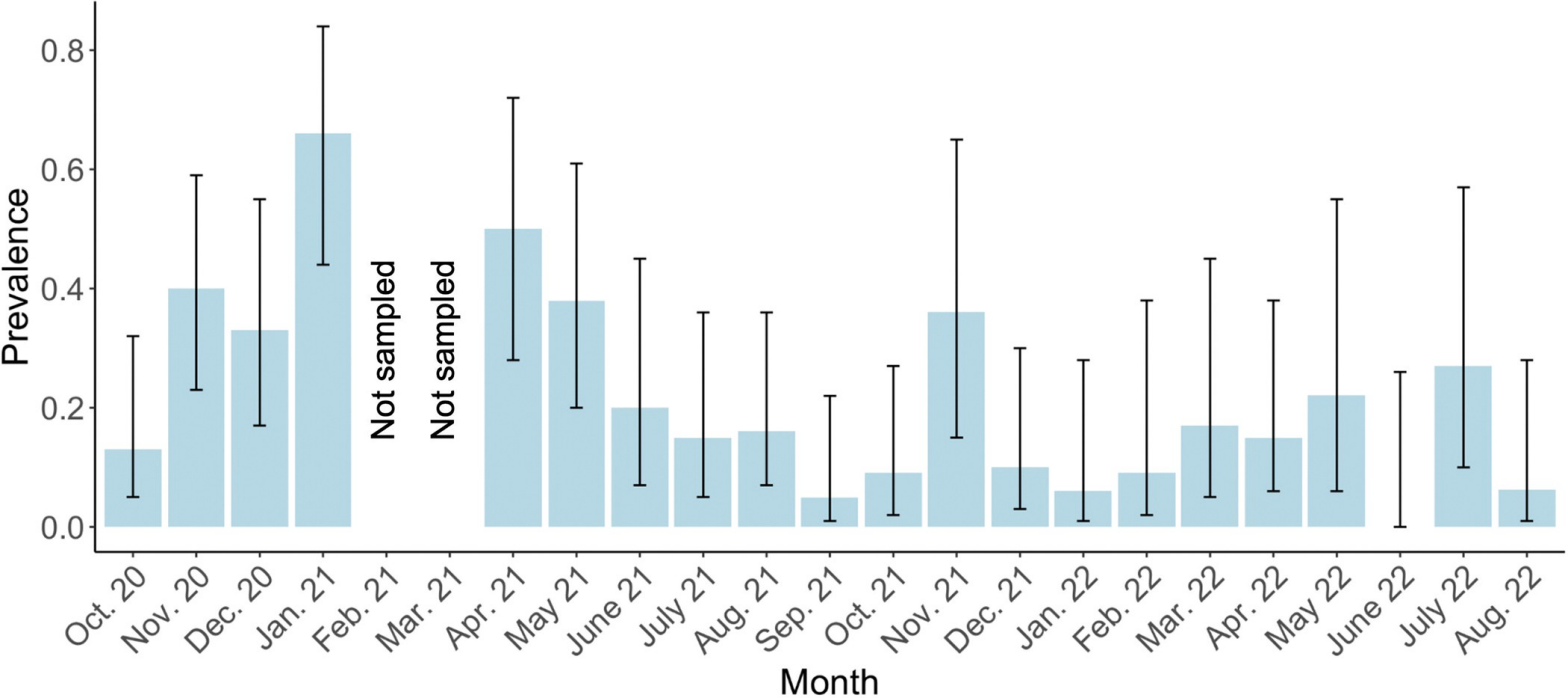
Seasonal variation of *T*. *gondii* DNA detection from feral cat feces (n = 362). Parasites were not detected in June 2022 (0% prevalence). Error bars correspond to 95% confidence intervals.

**Fig 2:**
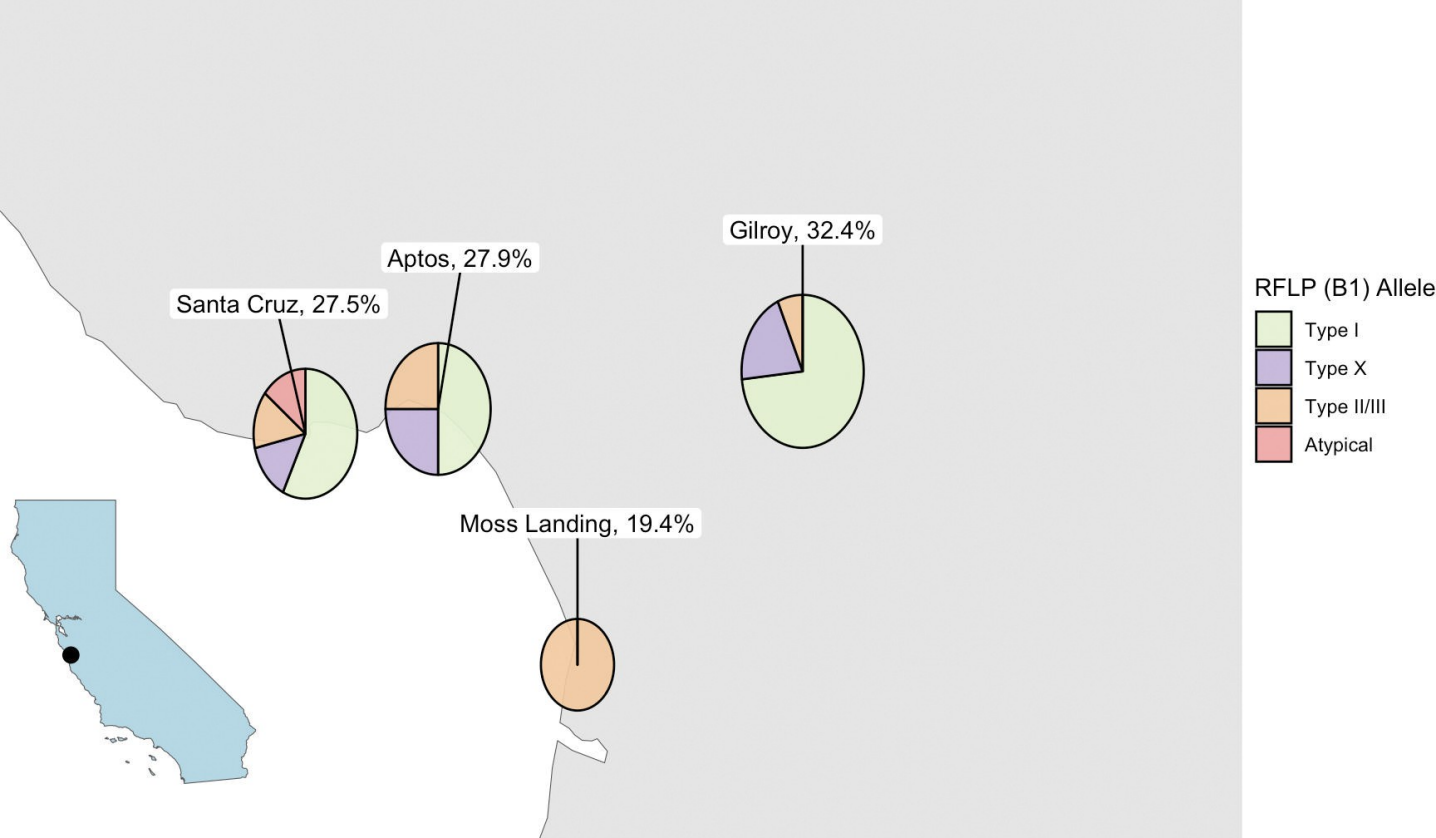
Prevalence of fecal samples that tested positive for *T*. *gondii* DNA in feral cats based in four colonies, and the relative distribution of parasite strains as characterized via RFLP at the B1 locus. Relative pie chart size is proportional to the overall *T*. *gondii* DNA prevalence. The California base map was extracted from Natural Earth using the rnaturalearth package [37].

The overall fecal *T*. *gondii* DNA prevalence was 25.9% but ranged from a low of 19.4% in Moss Landing to a high of 32.4% in Gilroy (Table 1). Differences between DNA detection at sites and year of sampling were unlikely to be due to chance (Table 2). Of the univariable explanatory variables, the number of adult cats at a sampling site (p = 0.063), season of sampling (wet/dry; p = 0.00045), sampling site (p = 0.13), number of kittens at a sampling site (p = 0.09), and presence of kittens at a sampling site (p = 0.013) met the significance threshold for further consideration in the multivariable Bayesian model. Kitten presence at a sampling site was selected over the number of kittens at a sampling site due to the lower p-value and biological significance of having any *T*. *gondii* naïve animals that have higher potential to shed oocysts into the environment. Although year was not significant (p = 0.79), we included both year and sampling site as random effects due to the level of clustering and repeat sampling, both spatially (sampling site) and temporally (year). The output of the Bayesian generalized linear mixed-effects model showed that prevalence varied seasonally, with 3.42 times higher odds of detection in the wet versus dry season (Fig 1 and Table 2). The presence of kittens at sampling sites was associated with 4.71 times higher odds of *T*. *gondii* DNA detection as compared to sites without kittens. The number of adult cats at the sampling site was associated with lower DNA detection (OR = 0.77). Spatially, more Type I and Type I variants were detected at the inland site versus coastal sites, which had higher proportions of Type X and X variant, as well as non-archetypal strains at the B1 locus (Fig 2).

**Table 2:** Results of Bayesian generalized linear mixed-effects regression model of *Toxoplasma gondii* DNA detected in feral cat feces (n = 362).

Variable	Odds Ratio (95% Credible Interval)
Random effect Site Year	1.39 (1.01, 3.78)* 1.47 (1.01, 5.05)*
Fixed effects Rainy season (wet vs dry) Number of adult cats at specific sampling site Kitten presence at sampling site (vs absence)	3.42 (1.97, 5.92)* 0.77 (0.62, 0.96)* 4.71 (2.03, 10.91)*

*Variables with lower and upper bounds of the 95% credible interval that do not cross 1 indicate significance.

**Fig 3:**
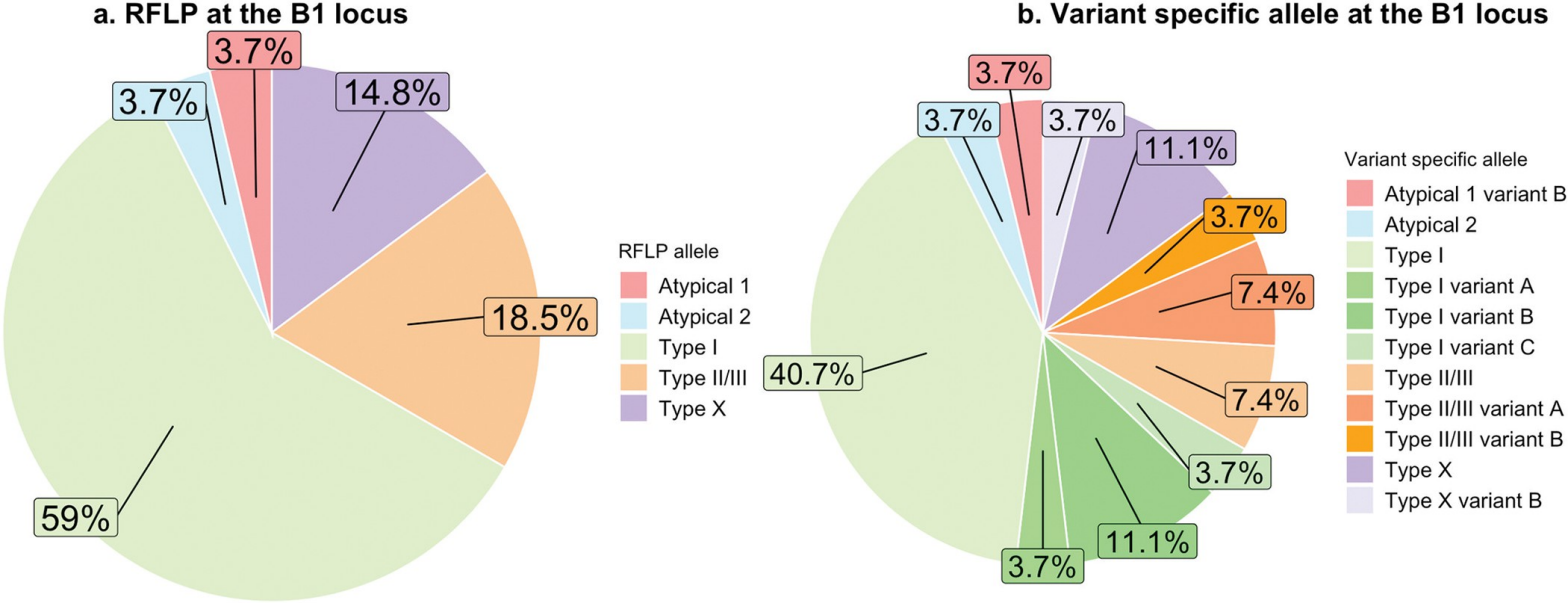
**A.** Distribution of *T*. *gondii* strains detected in feral cat feces (n = 27) as determined via virtual RFLP at the B1 locus. **3B.** Variant-specific sequence typing at the B1 locus of *T*. *gondii* strains detected in feral cat feces (n = 27). Variants were determined by the presence of one or more unique snps not located at restriction enzyme sites.

Of the 94 samples that tested positive for *T*. *gondii* at the ITS-1 locus, 27 were successfully amplified and characterized at the B1 locus (28.7%), and 13 of these were further characterized at one or more single-copy polymorphic loci (13.8%, S1 Table). Of the 27 samples that amplified at the B1 locus, the majority had alleles consistent with a Type I strain (59%), followed by Type II/III (18.5%), Type X (14.8%), and two separate non-archetypal cleaving patterns via virtual RFLP (Atypical 1, 3.7%; Atypical 2, 3.7%) (Fig 3A). Using a sequence analysis approach to characterize the B1 locus, these samples were further categorized into eight variant strains (Fig 3B) based on additional snps present in nucleotide sites not targeted by restriction enzymes [14]. Nine samples had novel snps when compared with reference and publicly available sequences and were categorized as variant, and a subset of these also had mixed base pairs at the B1 locus (as indicated by UPAC ambiguity codes S, Y, R, or M in Table 3). Five samples had unique snps at either the SAG1, SAG3, or L358 locus (S1 Table). However only one of these samples (Fecal no. 151) had confirmed novel snps at the L358 locus after re-sequencing. Samples subjected to RT-qPCR for attempted detection of mRNA from the SporoSAG gene did not yield visual amplification, indicating no detectable mRNA from sporozoites in these samples.

**Table 3:** Single nucleotide polymorphisms at the B1 locus in *Toxoplasma gondii* detected in feral domestic cat feces in central coastal California. IUPAC codes designate mixed nucleotide bases (S = C or G, Y = C or T, R = A or G, and M = A or C). A slash (/) indicates that different nucleotides were identified on different sequencing attempts and therefore no definitive base(s) could be reported at that position for some samples.

Reference strains (GenBank accession no.)	256	330	360	366* Xho	368* Xho	428	486	495	504* PmII	513	533	545	609	RFLP Type	MLST Type
ATCC strain RH (Type I)	A	T	C	T	C	T	T	G	G	T	A	C	A	I	I
ATCC strain ME49 (Type II)	A	T	C	**Y**	C	T	T	G	**S**	T	A	C	A	II/III	II/III
ATCC strain CTG (Type III)	A	T	C	**Y**	C	T	T	G	**S**	T	A	C	A	II/III	II/III
Type X (bobcat, KM243024)	A	T	C	T	C	T	T	G	C	T	A	C	A	X	X
Type X variant (sea otter, MK988572)	A	T	**S**	T	C	T	T	G	C	T	A	C	A	X	X variant A
CR34 (mussel, KM243022)	A	T	C	**C**	C	T	T	G	G	T	**C**	C	A	Atypical 1	Atypical 1 Variant A
**Fecal sample ID**															
76, 111, 118, 119, 150, 165, 253, 287, 349, 351, 389	A	T	C	T	C	T	T	G	G	T	A	C	A	I	I
77** (OQ850747)	A	T	C	T	C	T	T	G	G	T	A	C	**G**	I	I variant A
87, 129	A	T	C	T	C	T	T	G	S	T	A	C	A	II/III	II/III
92, 113, 354	A	T	C	T	C	T	T	G	C	T	A	C	A	X	X
128** (OQ850748)	A	T	C	T	T	T	T	G	C	T	A	C	A	X	X var B
151	A	T	C	T	C	T	T	G	**S/G**	T/Y	A	C	**R/G**	II/III	II/III variant A
155⌃	A	T	C	T	C	T	T	G	S	T	A	C	**R**	II/III	II/III variant A
159** (OQ850749)	A	T	C	T	C	T	T	G	G	T	A	C	**R**	I	I variant B
161	A	T	C	T	C	T	T	G	G	T	A	C	**R**	I	I variant B
163	A	T	C	**T/Y**	C	T	T	G	G	T	**A/M**	C	**R/G**	I	I variant B
188** (OQ850750)	**R**	**Y**	C	T	C	**Y**	**Y**	**R**	G	T	A	C	**G**	I	I variant C
204** (OQ850751)	A	T	C	**Y**	C	T	T	G	**S**	T	**M**	C	A	II/III	II/III variant B
279	A	T	C	**C**	C	T	T	G	G	T	C	**C/Y**	A	Atypical 1	Atypical 1 variant B
296	A	T	C	**C**	C	T	T	G	**C**	T	A	C	A	Atypical 2	Atypical 2

*Restriction enzyme cleaving site used for RFLP genotype designation.

^Re-amplification not attempted due to the lack of extracted DNA.

**Unique/new variants that were successfully sequenced at least twice with confirmation of new snps and submitted to GenBank.

## Discussion

We present evidence of a high prevalence (25.9%) of *T*. *gondii* DNA in feces from feral cats along the central California coastline near southern sea otter habitat. Lack of oocyst visualization in fecal samples that contain DNA from diverse strains of *T*. *gondii* is puzzling and may be due to several factors as discussed below. Notably, we detected non-archetypal alleles at the B1 locus for *T*. *gondii* DNA in cat feces, suggesting that these strains are present within feral cat populations that can contribute oocysts to watersheds draining runoff to sea otter habitat. Our results support previous studies highlighting the importance of free-ranging cats in coastal California as a source of *T*. *gondii* transmission to susceptible hosts (including people) in terrestrial, coastal, and marine environments [10,12,20].

**Fig 4:**
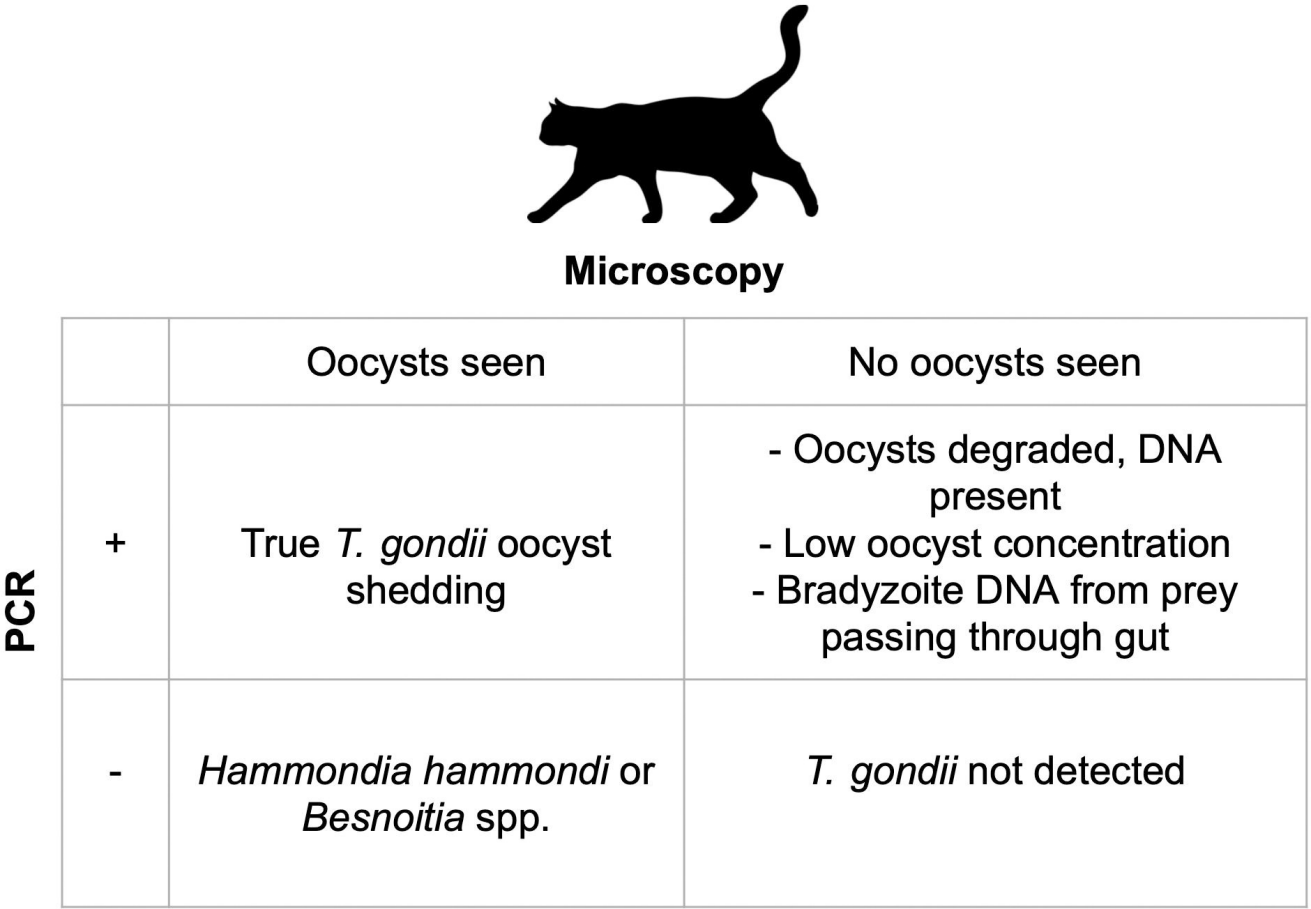
Interpretation of *T*. *gondii* detection in cat feces using microscopy and PCR methods.

Prior studies of *T*. *gondii* oocyst shedding in felids have used a combination of microscopy and PCR to confirm the identity of visualized parasites (Fig 4). Using microscopy alone, *T*. *gondii* cannot be discriminated from closely related apicomplexan oocysts that may occur in felid feces, namely *Hammondia hammondi* and/or *Besnoitia* spp. [41], so either PCR or bioassay is necessary to confirm the identity of *T*. *gondii*-like oocysts [42]. The use of PCR alone is challenging to interpret because the detection of *T*. *gondii* DNA in cat feces does not necessarily indicate oocyst shedding; rather, it is possible to detect DNA from tissue cysts in recently consumed infected prey passing through the gut and into the feces [28].

Other studies of *T*. *gondii* oocyst shedding have also contextualized their results in light of this critical methodological limitation [43,44]. While we applied molecular detection and microscopy in parallel, we did not observe any *T*. *gondii*-like oocysts, which may be due to procedural error, low concentrations of oocysts in feces, oocyst degradation in the environment before fecal collection, or true absence of oocyst shedding. While procedural errors in the fecal flotation method are possible, we consider these less likely given decades of experience in our laboratory with recovering and identifying apicomplexan oocysts, including *T*. *gondii*. In addition, our findings of other protozoan parasite oocysts in the same fecal samples (S2 Fig) suggests that the fecal flotation procedure for oocyst recovery was appropriate. Other recent publications in both owned domestic cats and free-ranging wild felids have described *T*. *gondii* DNA detection in the absence of oocyst visualization, which further highlights the previously mentioned issues for microscopy and a need for parallel confirmation [45, 46]. We acknowledge the limitations of PCR when applied on fecal samples due to inhibition of DNA amplification by organic compounds, amplification of non-target organisms, and cross-contamination between samples. Our assay quality control included means of identifying non-specific amplification (via sequence confirmation) and multiple negative controls to evaluate for any cross contamination; however, inhibition of parasite amplification in some samples may have occurred. We also attempted to identify oocyst mRNA using RT-qPCR [29], which was used to target a sporozoite-specific SAG protein mRNA. This technique could have allowed us to discriminate whether *T*. *gondii* detection was due to presence of viable oocysts vs bradyzoite DNA, however no samples were amplified using this method. It is important to note that the limit of detection for *T*. *gondii* mRNA is approximately 10 times higher than DNA based methods, so lack of amplification can either mean that no viable oocysts were present or that oocysts were present below the detection threshold [30].

Our findings provide evidence for frequent dietary or environmental exposure to *T*. *gondii*. The high prevalence of *T*. *gondii* DNA detected in feces indicates that cats frequently encounter *T*. *gondii* in prey, water, or soil. We found seasonal variation in *T*. *gondii* DNA detection in free-ranging cat feces, with higher likelihood of detection in the wet season. Seasonality of oocyst shedding and DNA detection may depend on location specific precipitation and temperature patterns. Among owned domestic cats in Germany, higher levels of *T*. *gondii* oocyst shedding were observed in summer and autumn (July-December) compared to winter and spring (January-June) [47]. Seasonal variation in oocyst shedding among owned domestic cats [48], seasonality of *T*. *gondii* detection in soil [20], and fluctuations in *T*. *gondii* infection in certain high-risk human populations (pregnant women [49,50]) have been reported but it is unclear exactly how seasonality affects *T*. *gondii* infection and oocyst shedding in free-ranging domestic cats. Free-ranging feral cats’ lifestyles and dietary habits make them one of the most likely felid groups to shed oocysts at any time of the year, partly because they are more likely to be co-infected with other parasites.

Though we did not see *T*. *gondii-*like oocysts, we did observe other parasites, including *Cystoisospora* spp. and *Toxocara cati* (S2 Table and S2 Fig), the latter of which is an underappreciated zoonotic pathogen [51]. After ingestion of larvated eggs by people, *T*. *cati* larvae can migrate to various organs and cause visceral larva migrans, a potentially severe and life-threatening disease in humans [52]. Knowing that feral cats are shedding zoonotic pathogens such as *T*. *cati* near human settlements is essential to convey to the general public, who may not be aware of this hazard. The presence of other parasites in feral cat feces can also inform another poorly understood aspect of *T*. *gondii* biology—the phenomenon of repeat shedding. Co-infection of *T*. *gondii* and other parasites like *C*. *felis* can induce repeat *T*. *gondii* oocyst shedding [53,54]. Though there was no association between visualizing other apicomplexans under microscopy and *T*. *gondii* DNA prevalence, detecting *Cystoisospora* spp. in feral cats suggests that this feral cat population may be more likely to experience repeat shedding of *T*. *gondii*. In this study, we were not able to evaluate repeated presence of *T*. *gondii* DNA in feces from the same individual cat over time because data on individual cat identity for each fecal sample were not attainable. We acknowledge this as an important limitation to the study that could lead to an overestimation of *T*. *gondii* DNA prevalence in the feral cat population at a given site.

Few studies have characterized the strains of *T*. *gondii* in free-ranging cat feces, and our study adds new data with relevant conservation implications. Importantly, we detected non-archetypal strains of *T*. *gondii* present in cat feces, including types that were previously associated with fatal toxoplasmosis in sea otters, Type X and X variants [14]. These *T*. *gondii* strains were also previously detected in tissues from nearby terrestrial domestic and wild felids, which points to the importance of assessing the terrestrial burden and source of *T*. *gondii*. Specifically, 4 out of 27 typed *T*. *gondii* samples from our study were Type X or X variants. Finding Type X and X variant *T*. *gondii* DNA in feces suggests that free-ranging feral cats are exposed to non-archetypal *T*. *gondii* strains, most likely in prey, that can result in shedding of more virulent *T*. *gondii* oocysts, which was previously documented among owned domestic cats [16,55]. A newly described virulent genotype for sea otters in California, COUG, has a Type I allele at the B1 locus, which was the dominant B1 allele in our feral cat fecal samples [56]. We were unable to further characterize *T*. *gondii* in feral cat feces due to low quality/quantity of DNA, so presence of the COUG genotype has not been confirmed in feral cats at this time. The presence of *T*. *gondii* DNA with the Type X allele (at the B1 locus) in feral cat feces may also be relevant to human health. A recent outbreak of meat-borne toxoplasmosis in Wisconsin hunters (presumed to be immunocompetent) resulted from consuming venison that was infected with *T*. *gondii* tissue cysts characterized as Type X [57].

Due to the limited quantity and quality of DNA we were unable to assign a ToxoDB genotype to our sequenced samples, as none amplified across more than 1–3 loci; our genotyping results were mostly limited to sequence analysis at the B1 locus. Only 13/27 samples that were sequenced at the B1 locus were successfully amplified and sequenced at one or more single copy loci commonly used in MLST or RFLP, and only two samples amplified at three loci. The B1 locus is a multi-copy gene, so observing mixed base snps at several sequence sites (S3 Fig) is challenging to interpret. These results may indicate either (i) presence of more than one strain in gut content (oocysts or bradyzoites) or (ii) polymorphisms within the different copies of the B1 gene (which is estimated to have 35 copies [31]) within a single strain (S3 Fig). For example, genotypes II and III have mixed bases at restriction cleaving sites that produce a predictable three band appearance following digestion with *Pml*I or *Xho*I, which allows for rapid discrimination from the more virulent Type I genotype [33].

**Fig 5:**
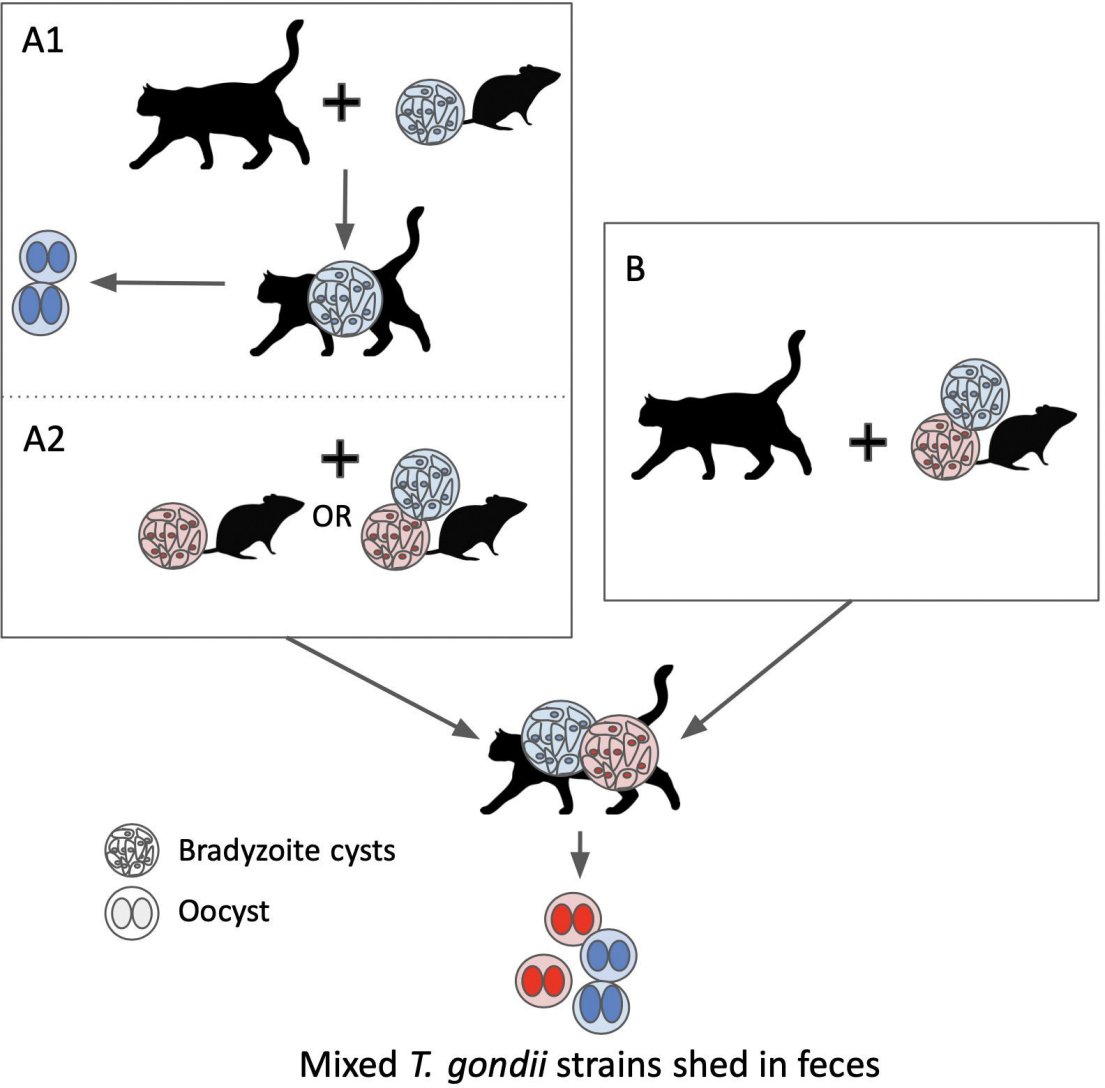
Mechanistic diagram showing how cats can be exposed to multiple *T*. *gondii* strains with subsequent shedding of mixed oocyst strains in feces. Red and blue indicate different *T*. *gondii* strain types in bradyzoite cysts and oocysts, and plus signs indicate consumption of *T*. *gondii* infected prey by felids. Mixed infection and shedding of more than one *T*. *gondii* strain can occur after animals are exposed to different parasite strains sequentially (Scenario A1 + A2), or simultaneously (Scenario B).

For the single copy gene SAG3 targeted by MLST in samples 279, 287, and 296, we had consistent Y (mixed C and T bases) at position 70 as confirmed by repeated sequencing (S1 Table). This observation likely represents the presence of more than one strain in a fecal sample from one animal, as it is not feasible for a single copy gene to have two different bases at a given nucleotide site. Mixed *T*. *gondii* genotype infections have previously been documented in felid tissues [18,58] but to our knowledge, the presence of two different parasite strains from feral cat feces has not been documented in North America. The presence of mixed strains in a cat fecal sample can indicate that a cat ate prey that was co-infected with two *T*. *gondii* strains and therefore has bradyzoites representing different strains passing through the gut, and/or a cat that is shedding *T*. *gondii* oocysts representing two different strains due to reactivation of previous infection following ingestion of a different genotype (Fig 5 Scenario A) or co-ingestion of two genotypes (that can result in sexual recombination) (Fig 5 Scenario B). Confirmation of mixed strains from feces is important as it demonstrates that diverse *T*. *gondii* genotypes are circulating in the environment and can increase the likelihood of oocyst re-shedding after cats encounter heterogenous strains.

Though not an aim of our study, our results demonstrate that both managed and unmanaged colonies had evidence of *T*. *gondii* DNA in feces. One of our sites was near a private home with several supplemental feeding areas, one was in a city park with supplemental feeding stations, and the other two colonies were near the beach without visible feeding stations despite extensive searching. Supplemental feeding could promote the survival and proliferation of larger colonies and increase predation of wildlife in an area [59,60]. Cat management, especially of unowned free-ranging cats, may be vital to wildlife conservation efforts, as measures like trap-neuter-release (TNR) are ineffective in the long term [61–63] and do not prevent domestic cats from killing native birds and small mammals via predation [64,65]. The aforementioned management techniques do not immediately address ongoing oocyst shedding and environmental contamination by feral cats. Keeping cats indoors can also be beneficial to cats, as many studies show lower seroprevalence of *T*. *gondii* [66] and longer life spans compared to outdoor animals [67]. It may be important to control current feral cat populations because natural mortality or artificial forces (TNR) may take too long to substantially reduce free-ranging cat population size in order to mitigate near-term impacts such as hunting and pathogen transmission to humans and native wildlife populations.

Our study demonstrates the potential of feral domestic cats as a population of felids that can contribute to the transmission of non-archetypal strains of *T*. *gondii*. Although pet cats can shed *T*. *gondii* oocysts, between 63 and 80.6% of pet cats in the United States are reported to be indoor-only [67,68]. Due to their large population sizes and association with human settlements, feral and free-ranging domestic cats are more important sources of parasites than their wild felid and pet cat counterparts in certain areas, and likely contribute a large proportion of the environmental oocyst load. Large populations of feral cats and *T*. *gondii* contamination near marine habitats can pose a risk for threatened and endangered marine mammals. Feral cat management has many potential benefits to cat welfare, public health, and wildlife conservation. Dialogue between diverse stakeholders is ultimately needed to find viable, sustainable solutions that prioritize cat welfare and wildlife health.

## Supporting information

S1 Table Results of attempted MLST sequencing of feral cat fecal samples that tested positive for *T*. *gondii* at the B1 gene. Samples where amplification and/or sequencing were unsuccessful are indicated with “—”.
(DOCX)

S2 Table Fecal samples with microscopy and PCR DNA detection of protozoan oocysts and helminth ova collected from four feral cat colonies in the greater Monterey Bay area, July 2020—August 2022. Parasite identify was determined using sequence analysis at the ITS-1 gene. There was no association between microscopy detection of other apicomplexa and *T*. *gondii* DNA detection.
(DOCX)

S1 Fig Number of monthly fecal samples collected from four feral cat colonies (Aptos, Gilroy, Moss Landing, and Santa Cruz) from July 2020—August 2022. No sampling was conducted in February and March 2021 due to COVID restrictions at that time.
(TIFF)

S2 Fig Brightfield microscopy images of helminth ova and apicomplexan oocysts or sporocysts identified via double centrifugation floatation in feral cat feces collected from the greater Monterey Bay area between 2020 and 2022. S2A. *Toxocara cati* ova (40X). S2B. *Cystoisospora* spp.-like oocyst (40X), S2c. *Sarcocystis* spp. sporocyst (40X).
(TIFF)

S3 Fig Chromatograms of *T*. *gondii* DNA sequences at the B1 locus for a feral cat fecal samples that had consistent mixed nucleotide polymorphisms with repeated sequencing trials (188) and a sample with inconsistent single and/or mixed nucleotide polymorphisms (151). Yellow bars under sequence nucleotide sites indicate locations of snps.
(DOCX)
