# Supplementary material for: High prevalence and diversity of Toxoplasma gondii DNA in feral cat feces from coastal California
Source: PLoS Negl Trop Dis. 2023 Dec 15;17(12):e0011829. doi: 10.1371/journal.pntd.0011829 (PMC10756541; doi:10.1371/journal.pntd.0011829)
Supplement: S1 Table — Samples where amplification and/or sequencing were unsuccessful are indicated with “—”. (DOCX) [file pntd.0011829.s001.docx]

**S1 Table.** Results of attempted MLST sequencing of feral cat fecal samples that tested positive for *T. gondii* at the B1 gene. Samples where amplification and/or sequencing were unsuccessful are indicated with “—”.

| ID | B1 | SAG 1 | 5'SAG2 | 3'SAG2 | alt SAG2 | SAG3 | BTUB | GRA6 | C22-8 | C292 | L358 | PK1 | Apico |
| --- | --- | --- | --- | --- | --- | --- | --- | --- | --- | --- | --- | --- | --- |
| 76 | Type I variant | — | — | — | — | — | — | — | — | — | — | — | — |
| 77 | Type I variant A | II/III | — | — | — | II/X | — | — | — | — | II | — | — |
| 87 | Type II/III | — | — | — | — | — | — | — | — | — | — | — | — |
| 92 | Type X | — | — | — | — | — | — | — | — | — | — | — | — |
| 110* | — | — | — | — | — | — | — | — | — | — | III | — | — |
| 111 | Type I | II/III | — | — | — | — | — | — | — | — | — | — | — |
| 113 | Type X | — | — | — | — | — | — | — | — | — | — | — | — |
| 118 | Type I | — | — | — | — | — | — | — | — | — | — | — | — |
| 119 | Type I | — | — | — | — | — | II | — | — | — | — | — | — |
| 128 | Type X var B | — | — | — | — | — | — | — | — | — | — | — | — |
| 129 | Type II/III | — | — | — | — | — | — | — | — | — | — | — | — |
| 150 | Type I | — | — | — | — | — | — | — | — | — | — | — | — |
| 151 | Type II/III var A | — | — | — | — | — | — | — | — | — | Unique** | — | — |
| 155 | Type II/III var A | — | — | — | — | — | — | — | — | — | — | — | — |
| 159 | Type I variant B | — | — | — | — | — | — | — | — | — | — | — | — |
| 161 | Type I variant B | — | — | — | — | — | — | — | II/X | — | — | — | — |
| 163 | Type I variant B | II/III | — | — | — | — | — | — | — | — | — | — | — |
| 165 | Type I | — | — | — | — | I | — | — | — | — | — | — | — |
| 188 | Type I variant C | — | — | — | — | — | — | — | — | — | — | — | — |
| 204 | Type II/III var C | — | — | — | — | — | — | — | — | — | — | — | — |
| 253 | Type I | — | — | — | — | — | — | — | — | — | — | — | II |
| 279 | Atypical 1 var B | — | — | — | — | II/X mixed^ | — | — | — | — | — | — | — |
| 287 | Type I | — | II/III/X (trimmed) | — | II/X | II/X mixed^ | — | — | — | — | — | — | — |
| 296 | Atypical 2 | — | — | — | — | II/X mixed^ | — | — | — | — | — | — | — |
| 349 | Type I | — | — | — | — | — | — | — | — | — | — | — | — |
| 351 | Type I | — | — | — | — | — | — | — | — | — | — | — | — |
| 354 | Type X | — | — | — | — | — | — | — | — | — | — | — | — |
| 389 | Type I | — | — | — | — | II/X | — | — | — | — | — | — | — |

*Sample 110 amplified at the B1 locus, however sequencing for this sample failed. MLST was performed and amplification and sequencing were successful at the L358 locus

**Sample 151 contained a snp confirmed via repeated sequencing at the L358 locus that produced a novel RFLP cleaving pattern (GenBank accession number OQ850752)

^Samples 279, 287 and 296 contained two distinct chromatogram peaks corresponding with C and T bases at nucleotide position 70. These mixed bases were confirmed in repeat sequencing and suggest the presence of more than one *T. gondii* strain present in fecal samples (Fig 5).
