# Supplementary material for: High prevalence and diversity of Toxoplasma gondii DNA in feral cat feces from coastal California
Source: PLoS Negl Trop Dis. 2023 Dec 15;17(12):e0011829. doi: 10.1371/journal.pntd.0011829 (PMC10756541; doi:10.1371/journal.pntd.0011829)
Supplement: S2 Table — Parasite identify was determined using sequence analysis at the ITS-1 gene. There was no association between microscopy detection of other apicomplexa and T. gondii DNA detection. (DOCX) [file pntd.0011829.s002.docx]

**S2 Table.** Fecal samples with microscopy and PCR DNA detection of protozoan oocysts and helminth ova collected from four feral cat colonies in the greater Monterey Bay area, July 2020 - August 2022. Parasite identify was determined using sequence analysis at the ITS-1 gene. There was no association between microscopy detection of other apicomplexa and *T. gondii* DNA detection.

| Parasite | Microscopy only  (*n* = 404) | PCR only  (*n* = 362) | Microscopy and PCR  (*n* = 362) |
| --- | --- | --- | --- |
| *Sarcocystis* spp. | 3 | 17 | 0 |
| *Cystoisospora* spp. | 13 | 8 | 7 |
| *Toxocara cati* | 31 | 0 | 0 |
