# Supplementary figures and images for: High prevalence and diversity of Toxoplasma gondii DNA in feral cat feces from coastal California
Source: PLoS Negl Trop Dis. 2023 Dec 15;17(12):e0011829. doi: 10.1371/journal.pntd.0011829 (PMC10756541; doi:10.1371/journal.pntd.0011829)

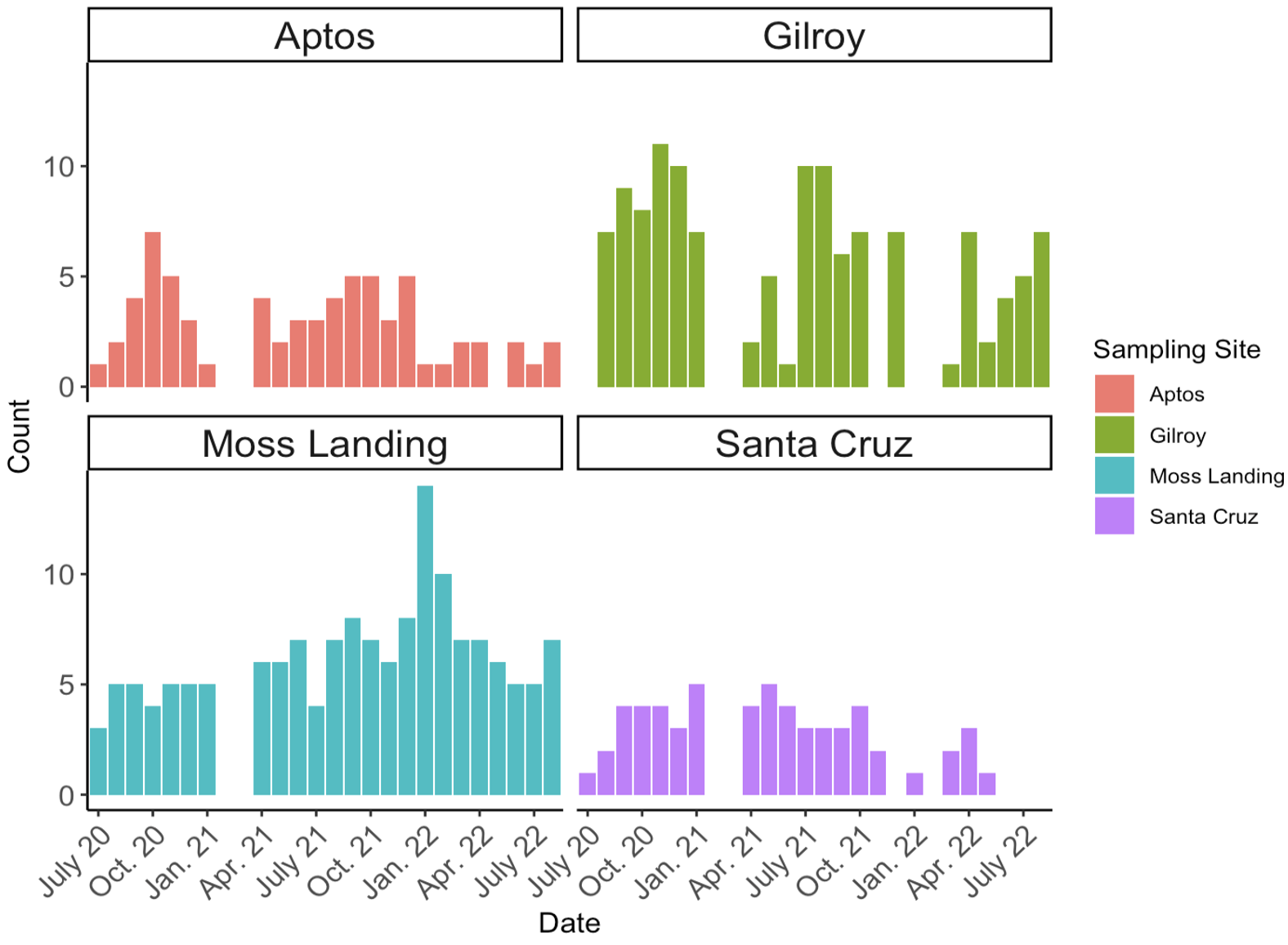

Supplement: S1 Fig — No sampling was conducted in February and March 2021 due to COVID restrictions at that time. (TIFF) [file pntd.0011829.s003.tiff]

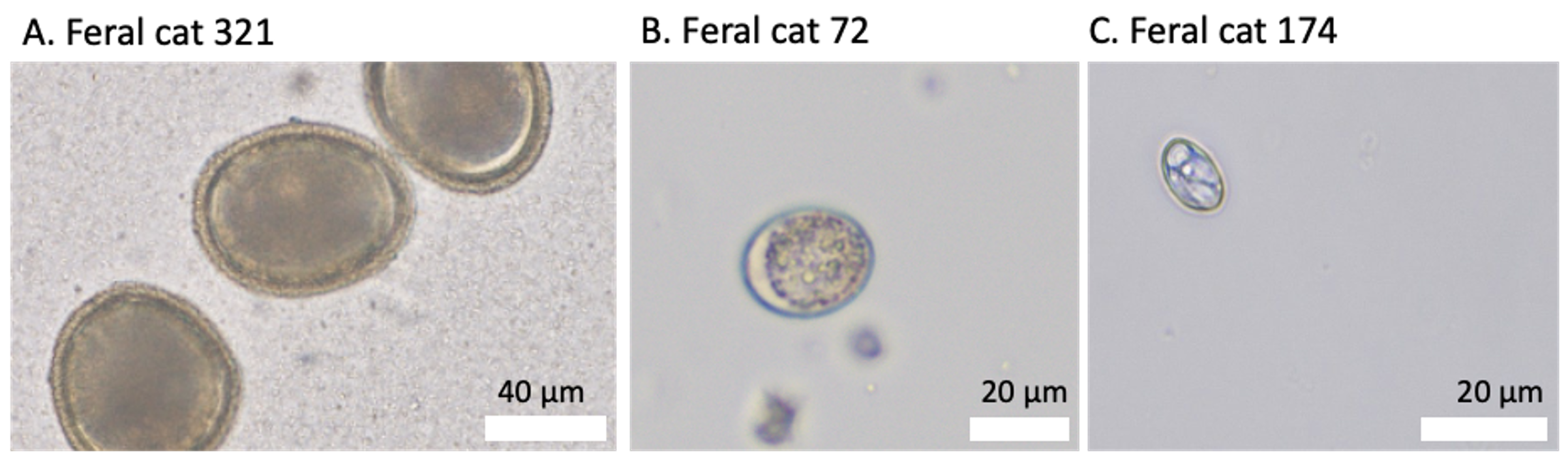

Supplement: S2 Fig — S2A. Toxocara cati ova (40X). S2B. Cystoisospora spp.-like oocyst (40X), S2c. Sarcocystis spp. sporocyst (40X). (TIFF) [file pntd.0011829.s004.tiff]
