# Supplementary material for: High prevalence and diversity of Toxoplasma gondii DNA in feral cat feces from coastal California
Source: PLoS Negl Trop Dis. 2023 Dec 15;17(12):e0011829. doi: 10.1371/journal.pntd.0011829 (PMC10756541; doi:10.1371/journal.pntd.0011829)
Supplement: S3 Fig — Yellow bars under sequence nucleotide sites indicate locations of snps. (DOCX) [file pntd.0011829.s005.docx]

**S3 Fig.** Chromatograms of *T. gondii* DNA sequences at the B1 locus for a feral cat fecal samples that had consistent mixed nucleotide polymorphisms with repeated sequencing trials (188) and a sample with inconsistent single and/or mixed nucleotide polymorphisms (151). Yellow bars under sequence nucleotide sites indicate locations of snps.

| Sample 188. Consistent snps at positions 228, 286, and 294. Confirmation of mixed bases with repeat sequencing in this sample supports a strain that contains polymorphic nucleotide bases across multiple copies of the B1 gene.  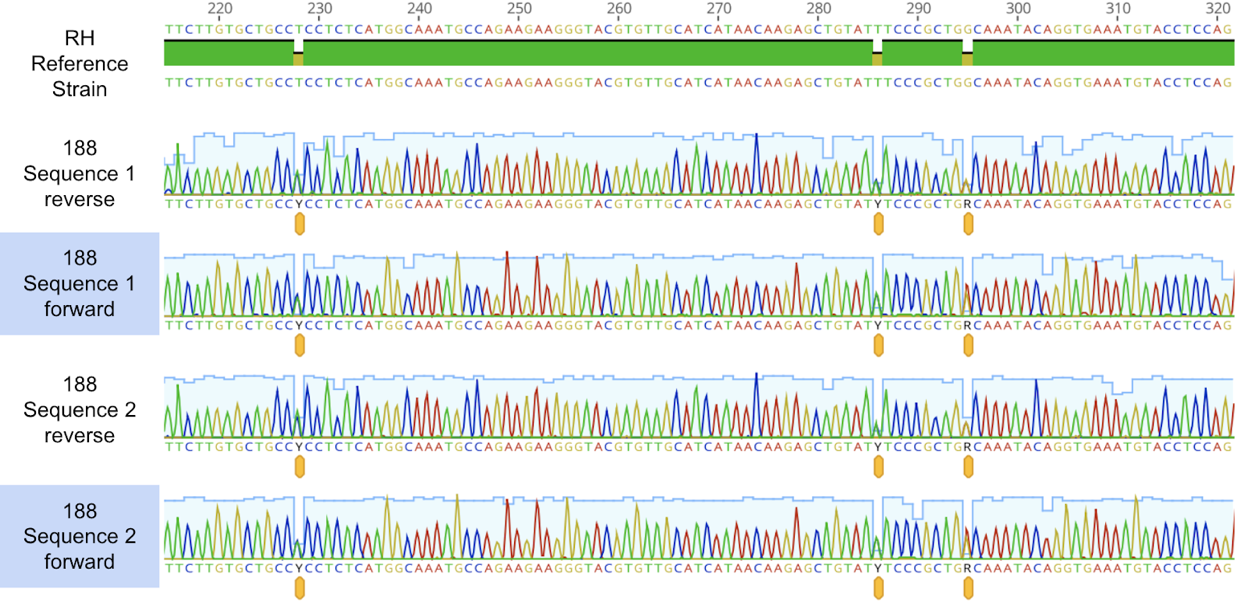 |
| --- |
| Sample 151. Inconsistent snps at positions 262, 273, 292, and 301. Lack of consistency in sequencing results (e.g. sample 151) suggests presence of more than one strain which could be the result of ingesting prey contaminated with different parasite strains or oocysts representing mixed genotypes (Figure 4).  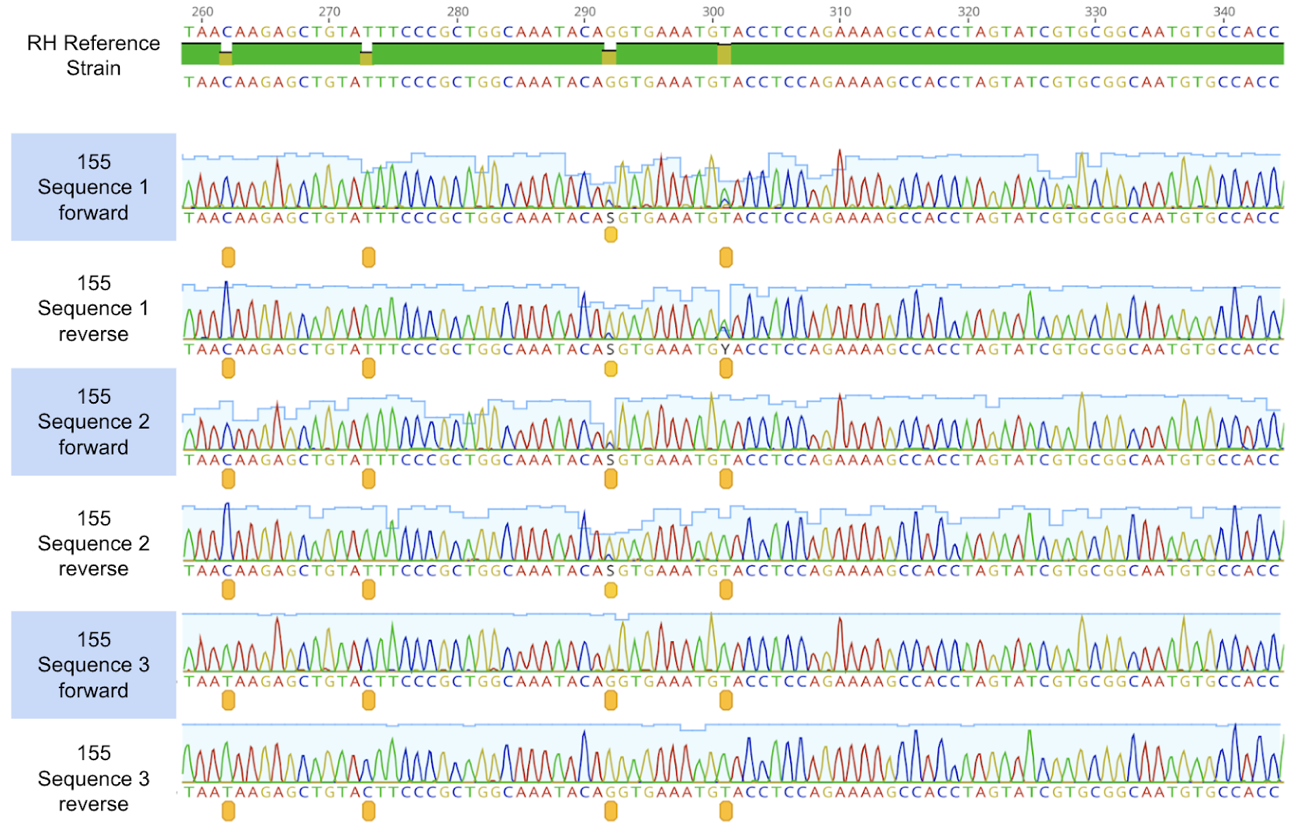 |
